# Characterization of Carnosine Effect on Human Microglial Cells under Basal Conditions

**DOI:** 10.3390/biomedicines11020474

**Published:** 2023-02-06

**Authors:** Giuseppe Caruso, Anna Privitera, Miriam Wissam Saab, Nicolò Musso, Salvatore Maugeri, Annamaria Fidilio, Anna Provvidenza Privitera, Alessandra Pittalà, Renaud Blaise Jolivet, Luca Lanzanò, Giuseppe Lazzarino, Filippo Caraci, Angela Maria Amorini

**Affiliations:** 1 Department of Drug and Health Sciences, University of Catania, 95125 Catania, Italy; 2 Unit of Neuropharmacology and Translational Neurosciences, Oasi Research Institute-IRCCS, 94018 Troina, Italy; 3 Department of Biomedical and Biotechnological Sciences, University of Catania, 95123 Catania, Italy; 4 Department of Physics and Astronomy “Ettore Majorana”, University of Catania, 95123 Catania, Italy; 5 Maastricht Centre for Systems Biology (MaCSBio), Maastricht University, 6200 MD Maastricht, The Netherlands

**Keywords:** carnosine, human microglia, inflammation, oxidative stress, energy metabolism

## Abstract

The activity of microglia is fundamental for the regulation of numerous physiological processes including brain development, synaptic plasticity, and neurogenesis, and its deviation from homeostasis can lead to pathological conditions, including numerous neurodegenerative disorders. Carnosine is a naturally occurring molecule with well-characterized antioxidant and anti-inflammatory activities, able to modulate the response and polarization of immune cells and ameliorate their cellular energy metabolism. The better understanding of microglia characteristics under basal physiological conditions, as well as the possible modulation of the mechanisms related to its response to environmental challenges and/or pro-inflammatory/pro-oxidant stimuli, are of utmost importance for the development of therapeutic strategies. In the present study, we assessed the activity of carnosine on human HMC3 microglial cells, first investigating the effects of increasing concentrations of carnosine on cell viability. When used at a concentration of 20 mM, carnosine led to a decrease of cell viability, paralleled by gene expression increase and decrease, respectively, of interleukin 6 and heme oxygenase 1. When using the maximal non-toxic concentration (10 mM), carnosine decreased nitric oxide bioavailability, with no changes in the intracellular levels of superoxide ion. The characterization of energy metabolism of HMC3 microglial cells under basal conditions, never reported before, demonstrated that it is mainly based on mitochondrial oxidative metabolism, paralleled by a high rate of biosynthetic reactions. The exposure of HMC3 cells to carnosine seems to ameliorate microglia energy state, as indicated by the increase in the adenosine triphosphate/adenosine diphosphate (ATP/ADP) ratio and energy charge potential. The improvement of cell energy metabolism mediated by 10 mM carnosine could represent a useful protective weapon in the case of human microglia undergoing stressing conditions.

## 1. Introduction

Microglia are myeloid cells and represent the primary component of the brain immune system. Microglia are best known as the immune cells of the brain, but emerging data indicate that they also influence brain development, synaptic plasticity, neurogenesis, memory, and mood under physiological conditions [1]. Both extension and retraction of microglial processes represent two types of motility required to regulate brain function, as well as to reach the sites of tissue damage [2]. The surveillance exerted by microglia is needed for various functions such as the identification of pathogens and the detection of brain damage [3], and the regulation of the activity of neurons and synapses [4]. Deviation of microglia from homeostasis, caused either by pro-inflammatory conditions (e.g., infections, stress, stroke, or neurodegenerative diseases) or microglial decline and senescence (e.g., aging or chronic unpredictable stress), are associated with impairments in neuronal plasticity and neurogenesis, often encountered in depressed patients [5,6]. Accordingly, some clinical phenotypes of depression may be considered as a microglial disease (microgliopathy), which should be treated through a personalized medical approach using inhibitors or stimulators of microglia response, depending on the microglial status of the patient [5]. Microglia also play a pivotal role in the inflammatory phenomena taking place at central nervous system (CNS) level [7]. Several studies have shown that activated microglia trigger the inflammatory process involved in various neurological disorders including Parkinson’s disease (PD), schizophrenia, Alzheimer’s disease (AD), and multiple sclerosis (MS) [8,9,10].

During the inflammatory process, activated microglial cells can perform phagocytosis leading to overproduction of reactive oxygen (ROS) and nitrogen (RNS) species, including nitric oxide (NO), this last compound mainly produced by upregulation of the inducible form of NO synthase (iNOS, also known as NOS2) [11,12]. The imbalance between oxidants and antioxidants is defined “oxidative stress” [13,14]. ROS, such as superoxide anion (O_2_^−•^), can react with RNS (e.g., NO) to form peroxynitrite (ONOO^−^), which is more toxic than the parent species, and is able to cause oxidative (inflammatory) damage involving DNA, proteins, and/or lipids [15,16], a common characteristic of neurodegenerative phenomena [17,18]. Therefore, the comprehension and possible modulation of the mechanisms regulating microglial response to injury/pathology are fundamental for the development of effective therapeutic strategies to counteract chronic neurodegeneration [11,19].

In recent years, several research studies have demonstrated that dietary nutraceuticals are able to mitigate microglial response, conferring neuroprotection in various neuropathological conditions such as injuries, infections, strokes, autism as well as neurodegenerative diseases including AD and PD [20,21,22]. Carnosine (β-alanyl-L-histidine) is a natural occurring dipeptide able to modulate brain cells activity through a multimodal mechanism of action [23]. It is synthetized by carnosine synthase 1 through an adenosine triphosphate (ATP)-consuming process, while its metabolism is regulated by the enzymes carnosinases (both cytosolic and plasmatic forms) [24,25]. This dipeptide is present in several mammalian tissues, with the highest tissue concentrations (mM levels) observed in skeletal and cardiac muscle [26] and in the brain [27]. There are different studies in the literature showing the ability of carnosine to exert a neuroprotective and anti-inflammatory activity through a multimodal mechanism of action including the detoxification of free radicals [28], the down-regulation of pro-inflammatory markers [29], as well as the modulation of immune cells such as macrophages and microglia [30], including the synthesis and the release of neurotrophins such as transforming growth factor beta-1 (TGF-β1) [31]. In addition, carnosine has shown the ability to modulate macrophage polarization and ameliorate cellular energy metabolism [30,32]. Despite that, research studies investigating the effects of carnosine on human microglia are missing.

Based on the above, in the present study, we first investigated the effects of increasing concentrations of carnosine on human microglia viability. We conducted these studies in HMC3, a well-known and established in vitro model to study inflammation and oxidative stress [33]. Once the maximum concentration not exerting toxicity was identified, it was used to investigate carnosine effects on the basal production of RNS/ROS (NO and O_2_^−•^) as well as on the variation of parameters representative of cellular energy metabolism.

## 2. Materials and Methods

### 2.1. Materials and Reagents

All materials and reagents were of analytical grade and supplied by Sigma-Aldrich (St. Louis, MO, USA) or Thermo Fisher Scientific (Waltham, MA, USA) unless differently specified. HMC3 (human microglia) cells (ATCC^®^ CRL-3304™), Eagle’s Minimum Essential Medium (EMEM), trypsin-EDTA solution, fetal bovine serum (FBS), and penicillin/streptomycin solution were purchased from American Type Culture Collection (ATCC, Manassas, VA, USA). High performance liquid chromatography (HPLC)-grade methanol, far-UV acetonitrile, and HPLC-grade chloroform were supplied by J. T. Baker Inc. (Phillipsburgh, NJ, USA). C-Chip disposable hemocytometers, employed for counting the cells, were obtained from Li StarFish S.r.l. (Naviglio, MI, Italy). QuantiTect Rev. Transcription Kit, QuantiTect SYBR Green PCR Kits, RNeasy Mini Kit, and QuantiTect Primer Assays were purchased from Qiagen (Hilden, NRW, Germany). 384-well plates were obtained by Roche Molecular Systems Inc. (Pleasanton, CA, USA). Eppendorf LoBind 1.5 mL Microcentrifuge Tubes PCR Clean as well as PCR tubes were both obtained from Eppendorf (Hamburg, HH, Germany). All water used in our study was Ultrapure (18.3 MΩ cm) (Milli-Q Synthesis A10, Millipore, Burlington, MA, USA).

### 2.2. Propagation and Maintenance of Cells

HMC3 cells were cultured in EMEM containing 10% (*v*/*v*) FBS, streptomycin (0.3 mg mL^–1^), penicillin (50 IU mL^–1^), sodium pyruvate (1 mM), GlutaMAX™ Supplement (1 mM), and MEM non-essential amino acids solution (1X) by using 25 or 75 cm^2^ polystyrene culture flasks. Cells were maintained in a humidified environment (37 °C, 5% CO_2_), and passaged every 3–5 days in order to avoid cell overgrowth.

### 2.3. Analysis of Cell Viability

One day before the experiment, HMC3 cells were harvested by using a trypsin-EDTA solution, counted with a C-Chip disposable hemocytometer, and plated in 96-well plates (8 × 10^3^ cells/well). The following day, cells were treated with increasing concentrations of carnosine (1, 10, and 20 mM) and incubated for 24 h in a humidified environment (37 °C, 5% CO_2_). Cell viability was measured through the well-known 3-(4,5-Dimethylthiazol-2-yl)-2,5-Diphenyltetrazolium Bromide (MTT) method [34]. Briefly, after the stimulation process, MTT solution (1 mg/mL in EMEM medium) was added to each well and cells were incubated (2 h, 37 °C). During the final step, dimethyl sulfoxide was used to melt the crystals, while the Synergy H1 Hybrid Multi-Mode Microplate Reader (Biotek, Shoreline, WA, USA) was used to read the absorbance at 569 nm. Data are the mean of four independent experiments (*n* = 4). Values were normalized with respect to control untreated HMC3 cells and are expressed as the percent variation of cell viability.

### 2.4. Analysis of Metabolites

The analysis of intracellular metabolites in deproteinized samples of HMC3 cells was performed by employing a HPLC method [35]. For this purpose, at the end of 24 h incubation in the presence or in the absence of carnosine (10 mM), cells were pelleted (at 4 °C) and washed twice with ice-cold PBS (pH 7.4). Cells were then deproteinized according to a well-established protocol based on organic solvent deproteinization allowing to measure acid labile as well as easily oxidizable compounds [36]. With the aim to simultaneously separate high-energy phosphates (e.g., ATP and guanosine triphosphate (GTP)), reduced glutathione (GSH), nicotinic coenzymes, malondialdehyde (MDA), and nitrite in the protein-free cell extracts we employed previously established ion pairing HPLC methods, in which tetrabutylammonium hydroxide was used as pairing reagent [37]. Separation was obtained by using a Hypersil C-18, 250 × 4.6 mm, 5 µm particle size column, provided with its own guard column, while the HPLC apparatus was composed of a SpectraSYSTEM P4000 pump system and a highly sensitive UV6000LP diode array detector, equipped with 5 cm light path flow cell. Both identification and quantification of each compound in chromatographic runs were obtained by comparing retention times, absorption spectra, and area of the peaks (high energy phosphates and nicotinic coenzymes: 260 nm; MDA: 266 nm; GSH and nitrite: 206 nm) belonging to the chromatographic runs of mixtures containing known concentrations of true ultrapure standard mixtures.

### 2.5. Gene Expression Analysis by Quantitative Real-Time PCR (qRT-PCR)

The concentration of total RNA, recovered from HMC3 under basal conditions (control) or cells treated with carnosine at the concentration of 10 mM for 24 h was determined by measuring the absorbance at 260 nm through a NanoDrop^®^ ND-1000 (Thermo Fisher Scientific, Waltham, MA, USA), while RNA quality was determined using Qubit^®^ 3.0 Fluorometer (Thermo Fisher Scientific). The reverse transcription of 1 μg of RNA (for each sample) was obtained by using QuantiTect Reverse Transcription Kit (Qiagen) according to manufacturer instructions, while the quantification of each cDNA sample loaded in a 384-well plate was obtained by employing a LightCycler^®^ 480 System (Roche Molecular Systems, Inc., Pleasanton, CA, USA). Table 1 reports the information related to the genomewide, bioinformatically validated primer sets (QuantiTect Primer Assays) employed for the gene expression analysis. 

The protocol used to perform sample amplification, fluorescence data collection, and sample quantification is the same as previously described [31,38]. Glyceraldehyde-3-phosphate dehydrogenase (GAPDH) was selected as housekeeping reference gene. As a negative control, a reaction in absence of cDNA (no template control, NTC) was performed.

### 2.6. Confocal Microscopy Analsysis

HMC3 cells were plated (2 × 10^4^ cells/well) on chambered coverslips (µ-Slide 8 Well Glass Bottom, Ibidi, Gräfelfing, MU, Germany) and allowed to grow overnight. The day after, cells were washed in PBS and stained with Hoechst 33342 (4 µM) [39] + 4-amino-5-methylamino-2′,7′-difluorofluorescein diacetate (DAF-FM DA) (10 µM) to measure NO intracellular levels [12,40], or MitoSOX Red (10 µM) to measure O_2_^−•^ intracellular levels [16,40], and left to incubate for a total of 60 min at 37 °C, 5% CO_2_.

Confocal fluorescence images were acquired on a Leica TCS SP8 confocal microscope, using an HC PL APO CS2 20X 0.75 NA objective lens and two hybrid photodetectors (Leica Microsystems, Mannheim, BW, Germany). Excitation wavelengths/emission bandwidths were the following: Hoechst 33342 (405/415-480), DAF-FM DA (488/500-570), and MitoSOX Red (488/550-680). The pinhole size was set to a value corresponding to 1 Airy Unit at a wavelength of 580 nm. Then, 2048 × 2048 pixel images were acquired with a pixel size of 189 nm. To minimize cross-talk, images of double stained cells, Hoechst 33342/DAF-FM DA or Hoechst 33342/MitoSOX Red, were acquired using the line sequential mode. Transmitted light images showing the morphology of the cells were acquired using the transmitted light detector (TLD) during excitation with the 488 nm laser.

The fluorescence intensity in the DAF-FM DA channel was evaluated in each image by selecting a region of interest (ROI) corresponding to the cells in the field of view and extracting the average value in the ROI. The fluorescence intensity in the MitoSOX Red channel was evaluated, in each image, by selecting a ROI corresponding to the cytoplasm of the cells and extracting the average value in the ROI. MitoSOX Red signal generated in the cell nuclei was excluded from the quantitative analysis. Nuclei were identified in the Hoechst 33342 channel. For each experimental condition, a minimum of 5 independent images obtained by 2 independent experiments were acquired and analyzed. For each experiment, one chambered coverslip (a total of 4 wells/condition) was considered.

### 2.7. Statistical Analysis

Statistical analysis was performed by using Graphpad Prism software, version 8.0 (Graphpad software, San Diego, CA, USA). Student’s *t*-test with Welch’s correction was used to assess the statistical differences between two experimental groups, while one-way analysis of variance (ANOVA), followed by Tukey’s post hoc test, was used for multiple comparisons. Only *p*-values of less than 0.05 were considered statistically significant. Data are always reported as the mean ± SD of at least 3 independent experiments. 

## 3. Results

### 3.1. Carnosine Does Not Affect Cell Viability at Concentrations up to 10 mM

**Figure 1:**
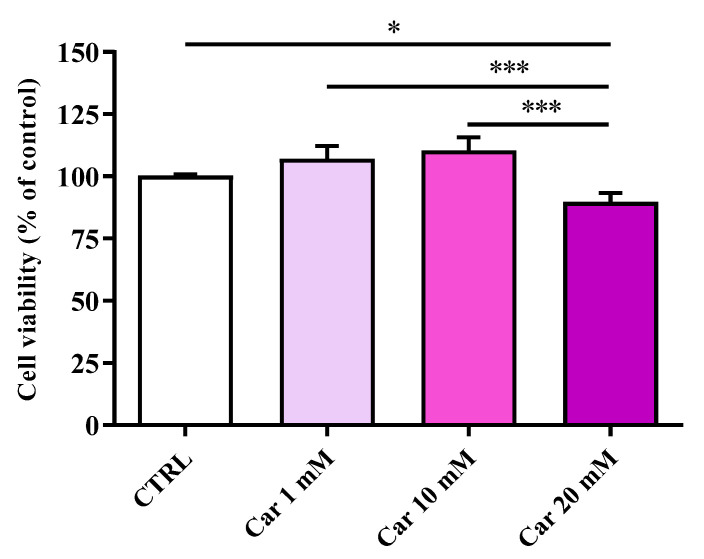
Cell viability in untreated HMC3 cells and in HMC3 cells treated with increasing concentrations of carnosine (Car) (1, 10, and 20 mM) for 24 h assessed by MTT assay. Data are the mean of four independent experiments (*n* = 4) and are expressed as the percent variation with respect to the cell viability recorded in untreated (CTRL) cells. Standard deviations are represented by vertical bars. * Significantly different, *p* < 0.05; *** significantly different, *p* < 0.001.

The first aim of the present study was to investigate whether carnosine exerts negative effects on HMC3 cell viability. Figure 1 shows the effects of increasing concentrations (1, 10, and 20 mM) of carnosine on the viability of human microglial cells.

Neither 1 mM nor 10 mM carnosine induced significant changes of cell viability compared to untreated (control) cells. A different effect was observed in the case of the maximum concentration (20 mM) of carnosine tested, that led to a cell viability % value that was significantly different to control cells (*p* < 0.05) as well as to 1 and 10 mM (*p* < 0.001).

### 3.2. Carnosine at 20 mM Increases IL-6 and Decreases HO-1 mRNA Expression Levels

**Figure 2:**
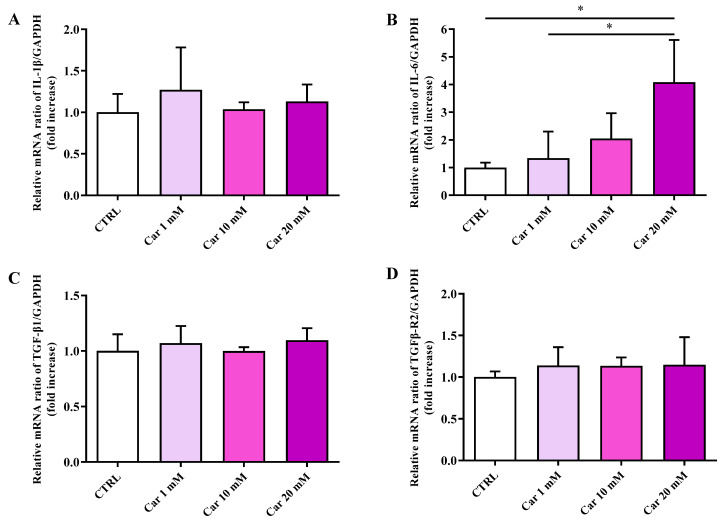
Measurement of (**A**) IL-1β, (**B**) IL-6, (**C**) TGF-β1, and (**D**) TGFβ-R2 mRNA expression levels in untreated HMC3 cells and in HMC3 cells treated with increasing concentrations of carnosine (Car) (1, 10, and 20 mM) for 24 h measured by qRT-PCR. The abundance of each mRNA of interest was expressed relative to the abundance of GAPDH-mRNA, as an internal control. qRT-PCR amplifications were performed in triplicate (*n* = 3). Standard deviations are represented by vertical bars. * Significantly different, *p* < 0.05.

In order to shed more light on the results obtained by carrying out the MTT assay (cell viability), we performed a gene expression analysis considering well-known markers of inflammation, such as interleukin (IL)-1β and IL-6 pro-inflammatory cytokines as well as the TGF-β1 anti-inflammatory cytokine and its receptor TGFβ-R2 [41,42], while inducible nitric oxide synthase (iNOS) and NADPH oxidase 2 (Nox-2) pro-oxidant enzymes [31,43] and heme oxygenase 1 (HO-1)/Kelch-like ECH-associated protein 1 (Keap-1)/nuclear factor erythroid 2–related factor 2 (Nrf2) [44,45] were selected as well-recognized markers of oxidative stress and antioxidant response, respectively. As clearly shown in Figure 2, none of the concentrations of carnosine tested were able to significantly modulate the mRNA expression levels of the pro-inflammatory cytokine IL-1β as well as that of the anti-inflammatory cytokine TGF-β1 and its receptor TGFβ-R2.

Of note, the highest concentration of carnosine tested (20 mM), the same able to induce a significant decrease of cell viability, led to a significant increase of IL-6 mRNA expression levels (*p* < 0.05 vs. CTRL and Car 1 mM).

**Figure 3:**
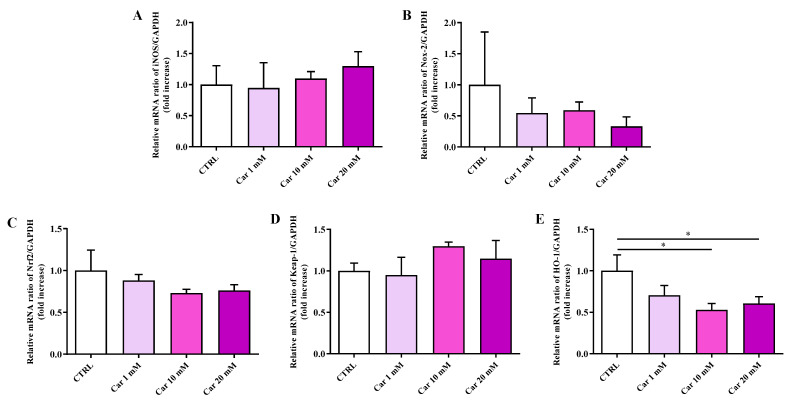
Measurement of (**A**) iNOS, (**B**) Nox-2, (**C**) Nrf2, (**D**) Keap-1, and (**E**) HO-1 mRNA expression levels in untreated HMC3 cells and in HMC3 cells treated with increasing concentrations of carnosine (Car) (1, 10, and 20 mM) for 24 h measured by qRT-PCR. The abundance of each mRNA of interest was expressed relative to the abundance of GAPDH-mRNA, as an internal control. qRT-PCR amplifications were performed in triplicate (*n* = 3). Standard deviations are represented by vertical bars. * Significantly different, *p* < 0.05.

As depicted in Figure 3, neither iNOS nor Nox-2 mRNA expression levels were significantly affected by the different concentrations of carnosine tested compared to the expression levels under basal conditions (CTRL).

We then considered the effects of carnosine on the mRNA expression levels of members of the antioxidant machinery represented by Nrf2, Keap-1, and HO-1. While no effects were observed in the case of Nrf2 and its inhibitor Keap-1, carnosine was able to significantly decrease HO-1 mRNA expression levels at the higher concentrations tested (*p* < 0.05 vs. CTRL).

From these sets of data, we were able to select the maximal carnosine concentration (10 mM) that can be used with human HMC3 microglial cells avoiding significant changes in cell viability and relative molecular alterations, represented by the increase of IL-6 and the decrease of HO-1 mRNA expression levels.

### 3.3. Carnosine Decreases NO Bioavailability

**Figure 4:**
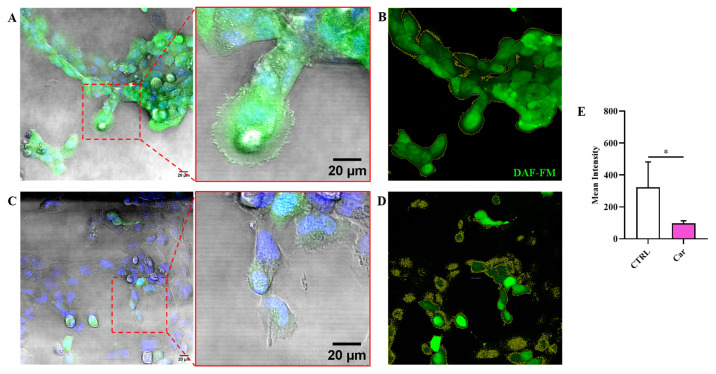
Representative confocal single z-plane images of (**A**) untreated HMC3 cells and (**C**) HMC3 cells treated with carnosine (Car) 10 mM for 24 h and stained with DAF-FM DA (green) and Hoechst 33342 (blue). The morphology of the cells is shown in the transmitted light detector channel (gray). Regions of interest (ROIs) used for the quantitative analysis of the images of (**B**) untreated and (**D**) treated cells. The ROIs are indicated by the yellow line and select the portion of the image corresponding to the cells. (**E**) Average value of DAF-FM DA fluorescence in HMC3 cells untreated or treated with carnosine. Data represent the mean of at least 5 images. Standard deviations are represented by vertical bars. * Significantly different, *p* < 0.05.

It is well-know that NO and O_2_^−•^, depending on their concentration, can play a key role in both physiological and pathological conditions. Figure 4 shows the significant decrese of NO levels as a consequence of the treatment of HMC3 cells with carnosine at the concentration of 10 mM (*p* < 0.05 vs. CTRL).

**Figure 5:**
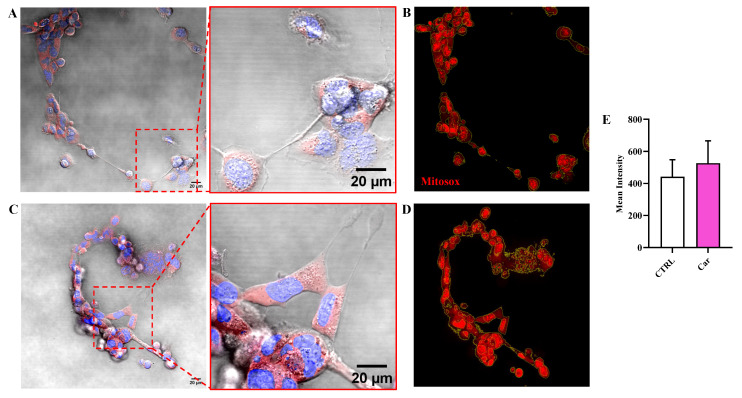
Representative confocal single z-plane images of (**A**) untreated HMC3 cells and (**C**) HMC3 cells treated with carnosine (Car) 10 mM for 24 h and stained with MitoSOX Red (red) and Hoechst 33342 (blue). The morphology of the cells is shown in the transmitted light detector channel (gray). Nuclei were removed from the MitoSOX Red channel for better visibility. Regions of interest (ROIs) used for the quantitative analysis of the images of (**B**) untreated and (**D**) treated cells. The ROIs are indicated by the yellow line and select the portion of the image corresponding to the cells. (**E**) Average value of MitoSOX Red fluorescence in HMC3 cells untreated or treated with carnosine. Data represent the mean of at least 5 images. Standard deviations are represented by vertical bars.

A different result was observed when measuring the fluorescence due the production of O_2_^−•^. In fact, differently from NO, the intracellular levels of O_2_^−•^ were not significantly modulated by the treatment of HMC3 cells with carnosine (Figure 5).

**Figure 6:**
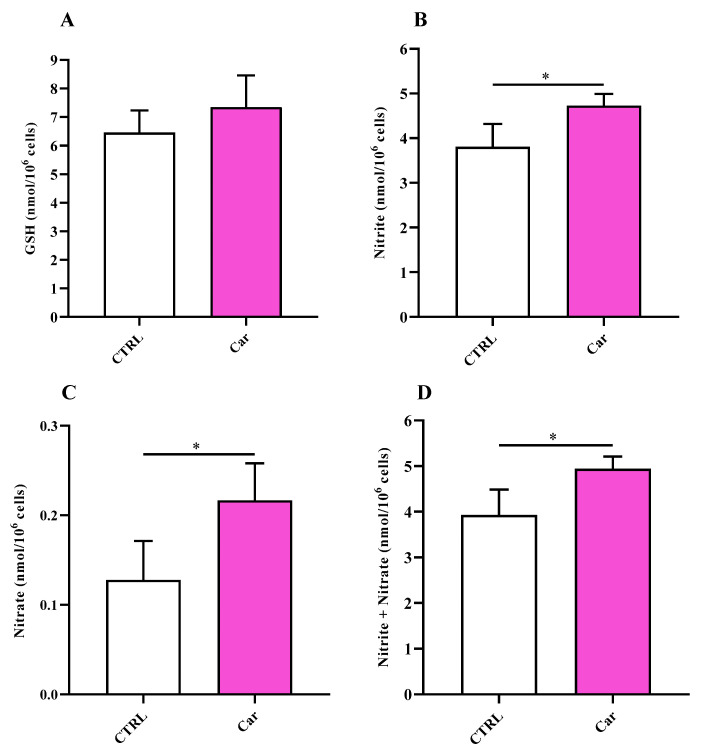
Values of (**A**) GSH, (**B**) nitrite, (**C**) nitrate, and (**D**) nitrite + nitrate determined in untreated HMC3 cells and HMC3 cells treated with carnosine (Car) 10 mM for 24 h by HPLC. Data represent the mean of 5 different experiments. Standard deviations are represented by vertical bars. * Significantly different, *p* < 0.05.

In order to investigate the mechanism regulating NO bioavailability in MHC3 microglial cells, we also measured the intracellular levels of NO metabolites (nitrite and nitrate) along with that of GSH, representing the main water-soluble antioxidant. Results reported in Figure 6A indicate that the treatment of HMC3 cells with carnosine did not cause significant changes in the concentration of GSH.

Differently from GSH, for which only a trend to increase was observed, the intracellular levels of both nitrite (Figure 6B) and nitrate (Figure 6C), as well as their sum (Figure 6D), increased as a consequence of carnosine exposure.

### 3.4. Carnosine Ameliorates Cellular Energy Metabolism Status

**Figure 7:**
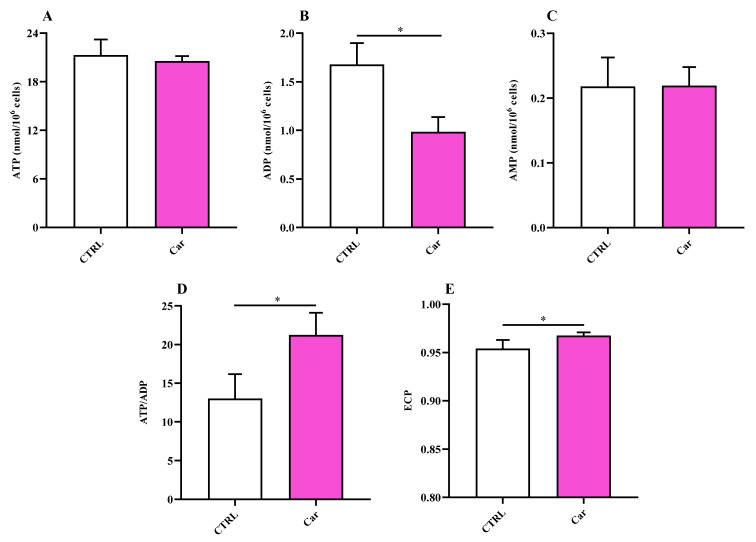
Values of (**A**) ATP, (**B**) ADP, (**C**) AMP, (**D**) ATP/ADP ratio, and (**E**) Energy Charge Potential (ECP = ATP + 1/2ADP/ATP + ADP + AMP) determined in untreated HMC3 cells and HMC3 cells treated with carnosine (Car) 10 mM for 24 h by HPLC. Data represent the mean of four different experiments. Standard deviations are represented by vertical bars. * Significantly different, *p* < 0.05.

Once the effects of carnosine on the modulation of parameters representative of inflammation, oxidative stress, and antioxidant response in human microglial cells was established, we moved our attention on the ability of this molecule to influence basal cellular energy metabolism status. The treatment of HMC3 with carnosine at the concentration of 10 mM for 24 h, whilst it did not cause changes in both ATP and adenosine monophosphate (AMP) levels (Figure 7A,C), produced a significant decrease in adenosine diphosphate (ADP) concentrations (Figure 7B), leading to a significant increase of the ATP/ADP ratio (Figure 7D; *p* < 0.05 vs. CTRL) that was accompanied by an increase of the energy charge potential (ECP) (Figure 7E; *p* < 0.05 vs. CTRL).

**Figure 8:**
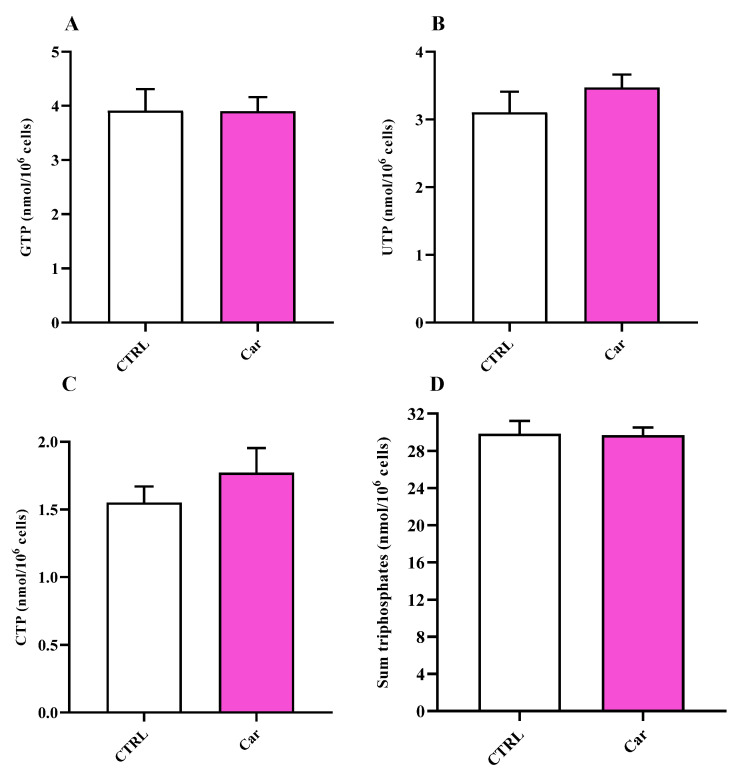
Values of (**A**) GTP, (**B**) UTP, (**C**) CTP, and (**D**) sum of triphosphates (ATP + GTP + UTP + CTP) determined in untreated HMC3 cells and HMC3 cells treated with carnosine (Car) 10 mM for 24 h by HPLC. Data represent the mean of four different experiments. Standard deviations are represented by vertical bars.

A trend of increased concentrations of uridine triphosphate (UTP) and cytidine triphosphate (CTP) (Figure 8), although not significant, was also found in HMC3 cells exposed to carnosine, whilst no differences in GTP and sum of triphosphates (ATP + GTP + UTP + CTP) concentrations were observed.

**Figure 9:**
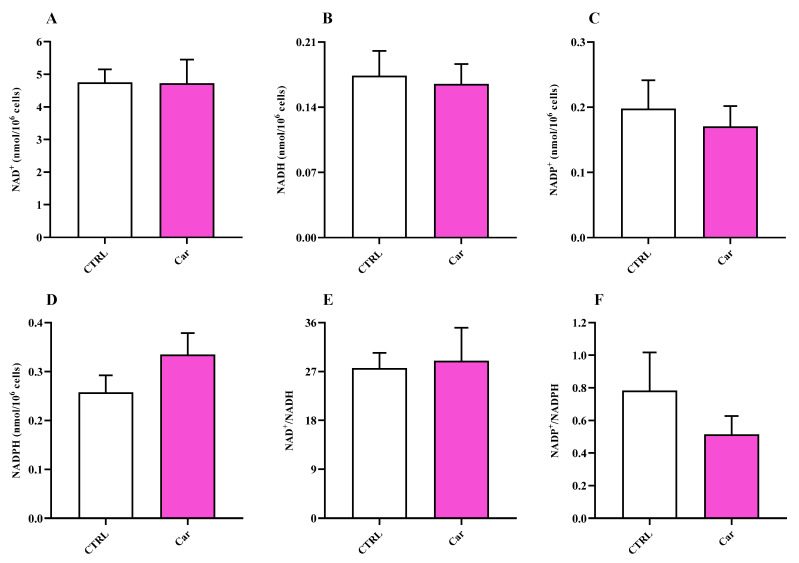
Values of (**A**,**C**) oxidized (NAD^+^ and NADP^+^) and (**B**,**D**) reduced (NADH and NADPH) nicotinic coenzymes determined in untreated HMC3 cells and HMC3 cells treated with carnosine (Car) 10 mM for 24 h by HPLC. The oxidized/reduced ratios (**E**,**F**) are also shown. Data represent the mean of 4 different experiments. Standard deviations are represented by vertical bars.

The measurement of nicotinic coenzyme values (Figure 9) suggested a capacity of carnosine to slightly influence nicotinamide-adenine dinucleotide phosphate (NADP) oxidized (NADP^+^) and reduced (NADPH) form levels, while no effects were observed on nicotinamide adenine dinucleotide (NAD) oxidized (NAD^+^) and reduced (NADH) forms.

**Figure 10:**
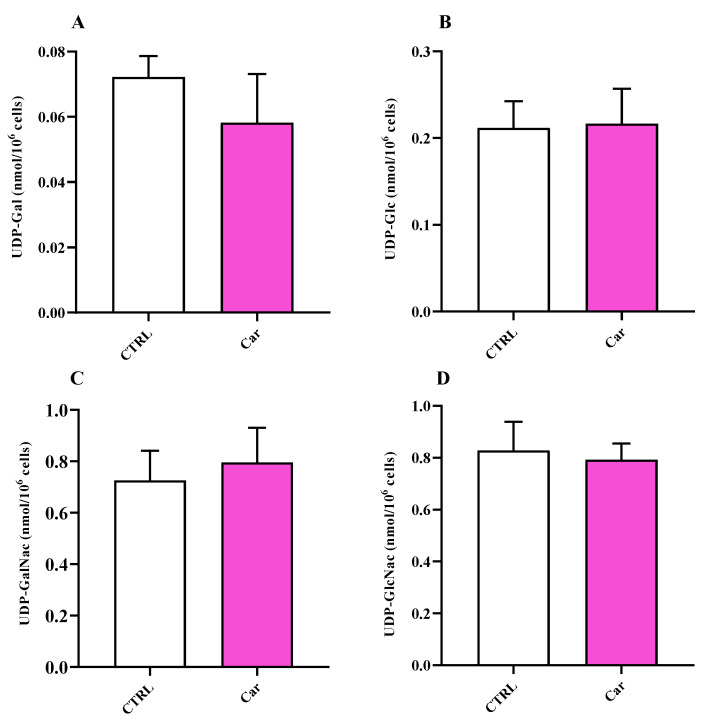
Values of (**A**) UDP-Gal, (**B**) UDP-Glc, (**C**) UDP-GalNac, and (**D**) UDP-GlcNac in untreated HMC3 cells and HMC3 cells treated with carnosine (Car) 10 mM for 24 h by HPLC. Data represent the mean of four different experiments. Standard deviations are represented by vertical bars.

Lastly, we analyzed the levels of uridine diphosphate (UDP)-derivatives, UDP-galactose (UDP-Gal), UDP-glucose (UDP-Glc), UDP-N-acetylgalactosamine (UDP-GalNac), and UDP-N-acetylglucosamine (UDP-GlcNac), in untreated HMC3 and in HMC3 exposed to carnosine. UDP-derivatives represent key mediators in ensuring the correct process of protein glycosylation indispensable for protein trafficking within and outside the cell [46,47]. As reported in Figure 10, no differences were observed between untreated and carnosine-treated HMC3 cells for any of the UDP-derivatives considered.

## 4. Discussion

The activity of microglia, the resident immune cells of the CNS, is fundamental for the regulation of numerous physiological processes such as brain development, synaptic plasticity, and neurogenesis [1,48,49]. As previously mentioned, the deviation from microglial homeostasis due to its response as a consequence of pro-oxidant/pro-inflammatory stimuli and/or decline and senescence can lead to different pathological conditions such as PD, AD, and MS [8,9,10]. Based on the above, the full understanding of microglia characteristics under basal physiological conditions as well as the possible modulation of the mechanisms related to its response are of utmost importance for the development of therapeutic strategies [11,19].

Numerous previous research studies have shown the importance of characterizing the effects of natural molecules/peptides on different cell types in the absence of stressing conditions as in the case of curcumin, resveratrol, oxyresveratrol, piceatannol, tea polyphenols, and carnosine [50,51,52,53,54,55,56,57,58,59,60]. Carnosine is a naturally occurring endogenous dipeptide with well-characterized antioxidant and anti-inflammatory activity [61]. This molecule has also shown the ability to “positively” modulate the activity of immune cells such as macrophages and microglia [30], leading to the enhancement of their antioxidant capacity as well as to the increased production of anti-inflammatory mediators [38].

With this in mind, in the present study, we first investigated the effects of carnosine on HMC3 cell viability. We focused our attention on carnosine concentrations (1, 10, and 20 mM) normally used in in vitro studies, with 20 mM representing the gold standard [56,62,63,64,65,66], a selection of concentration often sustained by the fact that it represents the highest concentration of carnosine that can be found at tissue levels. Interestingly, differently from 1 mM and 10 mM, the highest carnosine concentration, being 20 mM, induced a significant decrease of cell viability compared to control cells (Figure 1), that was paralleled by several molecular alterations corresponding to an increase of the mRNA expression level of the pro-inflammatory cytokine IL-6 (Figure 2B) as well as a decrease of the antioxidant mediator HO-1 (Figure 3E). This result could represent a very important finding not only for the present study, in which it allowed us to select the highest concentration of carnosine that can be used avoiding toxic effects, but also for future studies with human immune cells, and in particular HMC3 cells (the only human cell line currently available), wherein carnosine therapeutic potential will be tested. The HO-1 variation observed in the absence of Keap-1/Nrf2 modulation should not be surprising, since there are different research studies showing that HO-1 expression/induction can occur independently from the Nrf2 pathway [67,68,69,70]. The beneficial and protective effects of carnosine at 20 mM have been demonstrated in numerous studies employing murine immune cells (e.g., RAW 264.7) [38,63,65,71], but the use of the same concentration in HMC3 cells could lead to misleading results, representing the ratio between the multimodal mechanism of action of carnosine, being an antioxidant, anti-inflammatory, anti-aggregant, and neuroprotective molecule [6,23], and its ability to induce toxic effects when used at a very high concentration in these cells. In our experimental setup, carnosine treatment was able to decrease NO bioavailability (Figure 4). This is consistent with the well-characterized antioxidant activity of this dipeptide associated, from one hand, with its ability to directly interact with these species [54] and, from another hand, with the presence of the imidazole ring of histidine [27]. This result, along with that reported in Figure 6, is also in line with previous studies showing the ability of carnosine to decrease the levels of NO by increasing the rate of conversion of NO into its non-toxic end-product nitrite [32], without influencing its production, also measured as iNOS expression levels [38]. In particular, as previously described by performing electrospray ionization mass spectrometry and nuclear magnetic resonance spectroscopy analysis, a possible mechanism for the changes in NO levels in the presence of carnosine could be attributable to the ability of this dipeptide to form multiple adducts with NO (e.g., [Car+2NO]H^+^ and [Car+NO+NO_2_]H^+^, involving both its constituent amino acids (β-Ala and His). Of note, the ability of carnosine to increase the formation of nitrite levels is also in accordance with a study carried out by Takahashi et al. in which endothelial cells were exposed to increasing concentrations of carnosine, showing enhanced nitrite production in a dose-dependent manner [72]. This carnosine ability might be of relevance in contexts characterized by an overproduction of this RNS. In fact, it has already demonstrated that excessive levels of NO in immune cells are closely associated with different inflammatory diseases [73,74] and that when overproduced, NO can also react with O_2_^−•^ leading to the formation of ONOO^−^ [22]. Therefore, carnosine, by decreasing the levels of one of the substrates needed for the formation of ONOO^−^, diminishes the risks of peroxynitrite-mediated damages to fundamental macromolecules such as proteins and lipids [75]. It should also be taken into account that different studies have described the positive effects of nitrite supplementation [76,77] and then carnosine modulation of NO, increasing its biotransformation into nitrite, would not cause any adverse effect but might rather represent an additional positive pharmacological effect of this naturally occurring molecule. Based on our results (absence of carnosine-induced toxicity at 10 mM), the decrease in the physiological intracellular NO levels does not represent a negative event, at least under our experimental conditions; this is in agreement with other studies demonstrating that nitrites not only represent the principal storage source of NO, but are also able to mimic NO activity by acting on the same protein sites, in addition to the fact that physiological nitrites account for the basal levels of these modifications in vivo [78,79,80]. It is also well-known that most of the NO produced is widely assumed to be neutralized into its inert oxidation end products such as nitrites able to affect different biological responses such cyclic guanosine monophosphate (cGMP) production, cytochrome P450 activities, heat shock protein 70, and HO-1 expression in different tissues, hypoxic vasodilation, inhibition of mitochondrial respiration, and cytoprotection following ischemia/reperfusion [78,80].

It is well-known that exposure to ROS (e.g., O_2_^−•^) is not always biologically damaging, with cells responding to these species in a regulated way through the redox signaling [81]. It is worth mentioning that cell responses depend not only on the nature of the oxidant mediator, but also on the level of production and where it is produced. The intracellular levels of O_2_^−•^ were not significantly modulated by the treatment of HMC3 cells with carnosine (Figure 5). The absence of modulation O_2_^−•^ intracellular levels by carnosine under basal conditions could represent a positive event since differently from pathological conditions characterized by O_2_^−•^ overproduction, this ROS also represents an essential signaling molecule in normal physiology [82,83]. Furthermore, O_2_^−•^ radicals are widely produced under physiological conditions as a byproduct of oxygen metabolism and represent products of the metabolism of redox-active xenobiotics [81,84].

The significance of microglial energy metabolism in maintaining brain homeostasis has been recently discussed [85,86]. It has shown as the coordinated regulation of microglial inflammatory activation and energy metabolism, adapting to changes in brain energy homeostasis, play a key role in the function of these cells [86,87]. It is also worth underlining that under basal conditions, microglia primarily rely on oxidative phosphorylation (OxPHOS) for ATP production [86,87,88]. According to this scenario, we carried out for the first time the characterization of HMC3 microglial cells in terms of energy metabolites under both basal conditions and as a consequence of the presence of carnosine, that has already shown the ability to ameliorate immune cells energy status [30,88]. In our study, the treatment of HMC3 with carnosine at the concentration of 10 mM for 24 h led to a significant increase of the ATP/ADP ratio compared to control cells (Figure 7). The potential ability of carnosine to enhance ATP/ADP ratio, a measure of the cell phosphorylating capacity and energy generation through oxidative metabolism, may be of crucial relevance in the case of physical-chemical stimuli capable of negatively altering mitochondrial functions and of inducing oxidative stress through an excess of ROS production with decrease of intracellular antioxidant concentrations. No differences were instead found when measuring NAD^+^/NADH and NADP^+^/NADPH ratios between untreated and carnosine-treated HMC3 cells (Figure 9).

The energy pattern of untreated human microglia cells showed very high concentrations of ATP (Figure 7) [89,90,91] and other high-energy triphosphates (GTP, UTP, CTP; Figure 8), as well as values of the ATP/ADP ratio well above 10 (13.00 ± 3.17). Energy metabolism of these cell line is mainly based on mitochondrial OxPHOS coupled to efficient electron transfer chain (ETC) [86,87,88], as clearly indicated by the remarkably high value of the NAD^+^/NADH ratio (27.62 ± 2.81) (Figure 9). In fact, in a well-oxygenated living system (cells/tissues/organs), the NAD^+^/NADH ratio is from 15 to above (depending on the analytical method used to assay nicotinic coenzymes and on what cell/tissue/organ is considered); for instance, in cancer cells, that are more glycolytic even under basal conditions, the ratio is around 8–10 [92] and is around 30, 30–45, 150 in whole liver, brain, and heart homogenates, respectively [37,93,94]. In the case of oxygen deprivation or mitochondrial malfunctioning, cells/tissues/organs tend to counteract the consequent decrease in mitochondrially produced ATP by increasing glycolysis. Altogether, this leads to decrease in the NAD^+^/NADH ratio and cells rely their energy requirements mainly on glycolysis rather than on OxPHOS [37,92,93,95,96,97]. The values of the NAD^+^/NADH ratio (~27) along with that of ATP/ADP ratio (~13), generally ranging from 5 to 10 [37,98,99], measured in these human HMC3 microglial cells allowed us to affirm that these cells use the mitochondrial ETC coupled to OxPHOS for their energetic needs.

Conversely, compared to other cell lines in which values equal or higher than 2 were observed [92,100,101], the NAPD^+^/NADPH ratio has value lower than 1 (0.78 ± 0.23), suggesting that this cell line is characterized by a high rate of bio-synthetic reactions, possibly devoted to support lipid biosynthesis. Despite that, it should be taken into account that differently from microglia of the human brain, immortalized microglia (i.e., HMC3 cells) are not in their physiological environment and proliferating continuously, and thus characterized by different metabolic requirements [102].

## 5. Conclusions

In the present study, we provided evidence that carnosine, when used with human HMC3 microglial cells at a concentration of 20 mM, representing the gold standard in in vitro studies, led to a decrease of cell viability paralleled by an increase of IL-6 and a decrease of HO-1 in terms of gene expression. When using the maximal carnosine concentration avoiding significant changes in cell viability and relative molecular alterations (10 mM), carnosine decreased NO bioavailability, increasing its conversion into NO end-products nitrite and nitrate, without influencing O_2_^−•^ intracellular levels. In the present study, we also carried out, for the first time, the characterization of the energy metabolism of HMC3 microglial cells under basal (untreated) and carnosine exposure conditions, demonstrating that it is mainly based on mitochondrial OxPHOS coupled to efficient electron transfer paralleled by a high rate of biosynthetic reactions. Additionally, the exposure of HMC3 cells to carnosine led to a generalized amelioration of the microglia energy state, evaluated through the increase both in the ATP/ADP ratio and the ECP.

Our results suggest a therapeutic potential of carnosine and set the stage for future studies in which the neuroprotective activity of this dipeptide will be tested in the presence of pro-inflammatory/pro-oxidant stimuli in experimental models mimicking different neurodegenerative disorders, including PD and AD, characterized by microglia over-response, inflammation, oxidative stress, and energy unbalance.

## Figures and Tables

**Table 1:** The list of primers used for qRT-PCR.

Official Name ^#^	Official Symbol	Alternative Titles/Symbols	Detected Transcript	Amplicon Length	Cat. No. ^§^
interleukin 1, beta	IL1B	IL-1; IL1F2; IL1beta; IL1-BETA	NM_000576; XM_006712496	117 bp	QT00021385
interleukin 6	IL6	CDF; HGF; HSF; BSF2; IL-6; BSF-2; IFNB2; IFN-beta-2	NM_000600; XM_005249745	107 bp	QT00083720
nitric oxide synthase 2	NOS2	NOS; INOS; NOS2A; HEP-NOS	NM_000625; NM_153292	92 bp	QT00068740
cytochrome b-245 beta chain	CYBB	CGD; CGDX; NOX2; IMD34; AMCBX2; GP91-1; GP91PHOX; p91-PHOX; GP91-PHOX	NM_000397	124 bp	QT00029533
heme oxygenase 1	HMOX1	HO-1; HSP32; HMOX1D; bK286B10	NM_002133	99 bp	QT00092645
kelch like ECH associated protein 1	KEAP1	INrf2; KLHL19	XM_005260174; NM_012289; NM_203500; XM_005260173	109 bp	QT00080220
NFE2 like bZIP transcription factor 2	NFE2L2	NRF2; HEBP1; Nrf-2; IMDDHH	NM_006164	153 bp	QT00027384
transforming growth factor beta 1	TGFB1	CED; LAP; DPD1; TGFB; IBDIMDE; TGFbeta; TGF-beta1	NM_000660	108 bp	QT00000728
transforming growth factor beta receptor 2	TGFB2	AAT3; FAA3; LDS2; MFS2; RIIC; LDS1B; LDS2B; TAAD2; TBRII; TBR-ii; TGFR-2; tbetaR-II; TGFbeta-RII	NM_001024847; NM_003242; XM_006713316	108 bp	QT00014350
glyceraldehyde-3-phosphate dehydrogenase	GAPDH	G3PD; GAPD; HEL-S-162eP	NM_001256799; NM_002046; NM_001289745; NM_001289746	95 bp	QT00079247

^#^https://www.ncbi.nlm.nih.gov/gene/; ^§^https://www.qiagen.com/it/shop/pcr/real-time-pcr-enzymes-and-kits/two-step-qrt-pcr/quantitect-primer-assays/ (accessed on 15 December 2022).

## Data Availability

The data presented in this study are available on request from the corresponding author.
